# Ultrasound Doppler Flow in Patients With Chronic Midportion Achilles Tendinopathy: Is Surface Area Quantification a Reliable Method?

**DOI:** 10.1002/jum.15152

**Published:** 2019-11-14

**Authors:** Arco C. van der Vlist, Jasper M. Veen, Robert F. van Oosterom, Peter L. J. van Veldhoven, Jan A. N. Verhaar, Robert‐Jan de Vos

**Affiliations:** ^1^ Department of Orthopedic Surgery and Sports Medicine Erasmus University Medical Center Rotterdam the Netherlands; ^2^ Department of Sports Medicine The Hague Medical Center Antoniushove Leidschendam the Netherlands

**Keywords:** Achilles tendon, Doppler, reliability study, ultrasound

## Abstract

**Objectives:**

Ultrasound assessments of patients with chronic midportion Achilles tendinopathy include determining the degree of neovascularization using Doppler flow. A frequently used measure to quantify neovascularization is the modified Öhberg score. It is unknown whether the semiquantitative modified Öhberg score (0–4+) has higher reliability than a quantified measure of Doppler flow (0–100%). The purpose of this cross‐sectional study was to evaluate the interobserver reliability of the modified Öhberg score and a surface area quantification (SAQ) method for Doppler flow in patients with chronic midportion Achilles tendinopathy.

**Methods:**

Two observers examined the degree of Doppler flow independently using SAQ and the modified Öhberg score during a single consultation. The intraclass correlation coefficient, standard error of measurement, and minimal detectable difference were determined to evaluate the reliability and measurement properties of the SAQ method and the modified Öhberg score.

**Results:**

In total, 28 consecutive patients with chronic midportion Achilles tendinopathy participated. The intraclass correlation coefficient for interobserver reliability of the SAQ method was 0.81 (95% confidence interval, 0.58–0.91), compared to 0.64 (95% confidence interval, 0.45–0.81) for the modified Öhberg score. The standard error of measurement and minimal detectable difference values for the SAQ method were 2.9% and 8.0%, respectively, and for the modified Öhberg score, they were 0.55 and 1.53 points.

**Conclusions:**

The SAQ method shows good reliability to evaluate the degree of Doppler flow in patients with chronic midportion Achilles tendinopathy, and it overcomes the ceiling effect of the modified Öhberg score. Future research should focus on the relationship between the SAQ method and clinical outcomes and use this method to monitor treatment responses.

**Abbreviations**
AT: Achilles tendinopathy
CI: confidence interval
ICC: intraclass correlation coefficient
MDD: minimal detectable difference
PABAK‐OS: prevalence‐ and bias‐adjusted κ for ordinal data
PDUS: power Doppler ultrasound
RCT: randomized clinical trial
SAQ: surface area quantification
SEM: standard error of measurement
US: ultrasound
VISA‐A: Victorian Institute of Sport Assessment–Achilles

Chronic midportion Achilles tendinopathy (AT) is a degenerative condition of the Achilles tendon that most often occurs in running sports.^1^, ^2^, ^3^ Up to 52% of running athletes have AT at least once in their lifetimes.^1^ The clinical diagnosis AT is based on a combination of local Achilles tendon pain, swelling of the Achilles tendon, and an impaired load‐bearing capacity.^4^, ^5^

Ultrasound (US) is frequently used to verify the diagnosis of AT. One of the features that have been reported in a substantial number of articles is the prevalence of US Doppler flow. Ultrasound Doppler flow indicates neovascularization, which is the formation of new small blood vessels within and around tendons.^6^ This process is driven by the production of vascular endothelial growth factor, which also stimulates nerve growth alongside the neovascularization.^7^, ^8^ It is hypothesized that these newly formed nerve structures are a contributor or the cause of the pain in chronic tendinopathy.^6^ Since these nerve structures cannot be visualized with US, US Doppler flow is being used as a marker for the amount of newly formed nerve structures.

Increased US Doppler flow can be determined by both color Doppler ultrasound and power Doppler ultrasound (PDUS). Power Doppler ultrasound is the preferred method to use, since it is more sensitive to detect blood flow and is less operator dependent.^9^ Increased Doppler flow is present in 47% to 100% of symptomatic Achilles tendons compared to 0% to 50% of asymptomatic tendons.^6^, ^10^, ^11^, ^12^, ^13^, ^14^, ^15^, ^16^, ^17^, ^18^ Most of the studies used the modified Öhberg score to quantify the amount of Doppler flow, which has shown higher reliability compared to the original Öhberg score.^19^ The modified Öhberg score runs from 0 to 4 + .^4^, ^20^ A higher score indicates more Doppler flow in the peritendinous and intratendinous tissues.

The interobserver reliability of the modified Öhberg score varies from moderate to perfect.^10^, ^12^, ^19^ Some studies described other disadvantages of the modified Öhberg score. It is considered to have weak applicability to measure higher amounts of Doppler flow (called the ceiling effect), and the scoring system is operator dependent, since the assessment of the score is difficult.^11^, ^20^ To make the assessment less difficult, it has been suggested to reduce the modified Öhberg score to a 4‐point scale by combining the 3+ and 4+ categories.^10^, ^12^ This will, however, further increase the ceiling effect of this method. Therefore, reliable alternatives for the modified Öhberg score with a quantitative approach are warranted. The surface area quantification (SAQ) method was introduced by Boesen et al^21^ to overcome these limitations and has been used by several other research groups.^22^ This method aims to determine the percentage of color pixels within the Achilles tendon, the peritendinous region, or both. This enables a more quantitative analysis and potentially increases the accuracy of the measurement in a research setting. As the analysis is directly accessible and easy to perform, implementation in the clinical setting would also be feasible if medical device manufacturers were to implement this method as a real‐time application on their US machines. Both previous studies that investigated the SAQ method standardized the color Doppler settings for all examinations to increase reliability.^21^, ^22^ The reliability of such a standardized method is, however, currently unknown because previous studies did not assess reliability measures.

Our primary aim was to compare the reliability of the SAQ method with the modified Öhberg method in patients with AT by determining the intraclass correlation coefficient (ICC) between 2 observers. We hypothesized that the ICC of the SAQ method would be considerably higher compared to the semiquantitative modified Öhberg method. Secondary aims were to evaluate the correlation between both methods to test whether the same feature (amount of Doppler flow) was being measured and to evaluate the standard error of measurement (SEM) and minimal detectable differences (MDD) of these methods.

## Materials and Methods

### *Study Design*

This cross‐sectional study was conducted as a part of an ongoing double‐blind, placebo‐controlled randomized clinical trial (RCT). The aim of this RCT was to evaluate the effect of a high‐volume injection in patients with chronic midportion AT (http://clinicaltrials.gov identifier NCT02996409). Written informed consent was obtained before participation for all patients and also for the cross‐sectional part of this study. The protocol of the study was approved by the regional Medical Ethical Committee (registration number 14‐100).

### *Setting and Participants*

This cross‐sectional study was performed at the Department of Sports Medicine in a large district general hospital (The Hague Medical Center). The first 32 consecutive included patients who participated in the RCT were asked to take part in this cross‐sectional study from April 2017 to November 2017. Inclusion criteria were the presence of a chronic midportion AT for at least 2 months, the completion of an exercise training program for at least 6 weeks with an unsatisfactory outcome, age between 18 and 70 years, and the presence of Doppler flow on PDUS imaging. The diagnosis was established on the basis of a clinical examination (local tendon pain, swelling of the Achilles tendon 2–7 cm proximal to its calcaneal insertion, and an impaired load‐bearing capacity) by a physician with experience in sports medicine (15–20 years). As AT is considered a clinical diagnosis in principle, grayscale US was not used to verify the diagnosis.^3^ Main exclusion criteria were a clinical suspicion of other musculoskeletal disorders (insertional AT, inflammatory systemic disorders, and quinolone‐, corticosteroid‐ or statin‐induced tendinopathy), a previous Achilles tendon rupture or surgery, the inability to perform an exercise program, and a medical condition that would affect the safety of the injection (eg, peripheral vascular disease or the use of anticoagulant medication). Detailed information regarding all exclusion criteria is provided in the trial registration. In cases of bilateral symptoms, only the most affected tendon was included in the study.

### *Test Methods*

#### *Demographic Details*

Demographic characteristics of the study population were collected at baseline. We collected the following characteristics: age, sex, body mass index, duration of symptoms, Victorian Institute of Sport Assessment–Achilles (VISA‐A) score, primary sport, and activity level. The VISA‐A questionnaire consists of 8 questions and covers 3 domains: pain, activity, and function. Scores vary from 0 to 100, where 100 indicates an asymptomatic person, and 0 is defined as maximum pain, no activity, and no function. The activity level was determined by the researcher as recreational (1 or 2 sports activities per week), competitive (≥3 sports activities per week), or professional (≥3 sports activities per week at a national level).

#### *Ultrasound*

Two observers analyzed the Achilles tendons of the included patients independently during a single consultation. The PDUS was performed by 1 observer, while the other observer was not present in the room to maintain blinding. Both observers (1 PhD candidate [A.C.v.d.V.] and 1 research student (J.M.V.]) were trained to perform US measurements of the Achilles tendon with greater than 20 training hours and at least 10 patients before the start of the study. Ultrasound examinations for this study were performed during one of the follow‐up visits after inclusion in the RCT. Before the US examination was performed, all patients climbed 2 stairs to reach the examination room. No specific instructions were provided about activities before the consultation (eg, sports activities the day before). The patient was placed in a prone position on the examination table, and the ankle was placed over the table in a relaxed position. A Pro Focus type 2202 US scanner (BK Medical, Herlev, Denmark) with a type 8811 5–12‐MHz linear transducer was used to perform US measurements. Neovascularization was detected by PDUS with predefined settings: mechanical index, 1.28; thermal index, 1.2; pulse repetition frequency, 1.0 kHz; and gain, 50%. These settings were determined before the start of the study according to the optimization suggestions by Yang et al.^9^ Depth was standardized for every patient at 3.0 cm, and the color box size during the PDUS examination at 4.6 cm^2^ (depth, 1.7 cm; width, 2.7 cm). This color box size was chosen to measure the maximum intratendinous Doppler flow and peritendinous flow in the Kager triangle. Peritendinous flow was included to assess the same regions of interest for both the modified Öhberg score and the SAQ method.

The transducer was placed perpendicular to obtain a sagittal view of the Achilles tendon at the most painful part on palpation. The upper limit of the color box was placed on the dorsal side of the tendon. Pressure from the transducer was kept to a minimum to prevent occlusion of neovascularization.^16^, ^23^ Both observers screened the tendon for the area of maximum Doppler flow during the preparation phase for 1 minute. The transducer was gently moved to medial and lateral over the area where Doppler flow was present. When the location of maximum Doppler flow was identified, a 20‐second video was recorded. The modified Öhberg score was determined dynamically during the US examination according to previous studies.^10^, ^16^, ^24^ When the first observer completed the US examination, the second observer directly performed the examination to keep the time between the examinations to a minimum. The patient remained in the same prone position and was blinded to the outcome of the first observer to standardize for possible confounders.

#### *Analysis Using SAQ*

**Figure 1:**
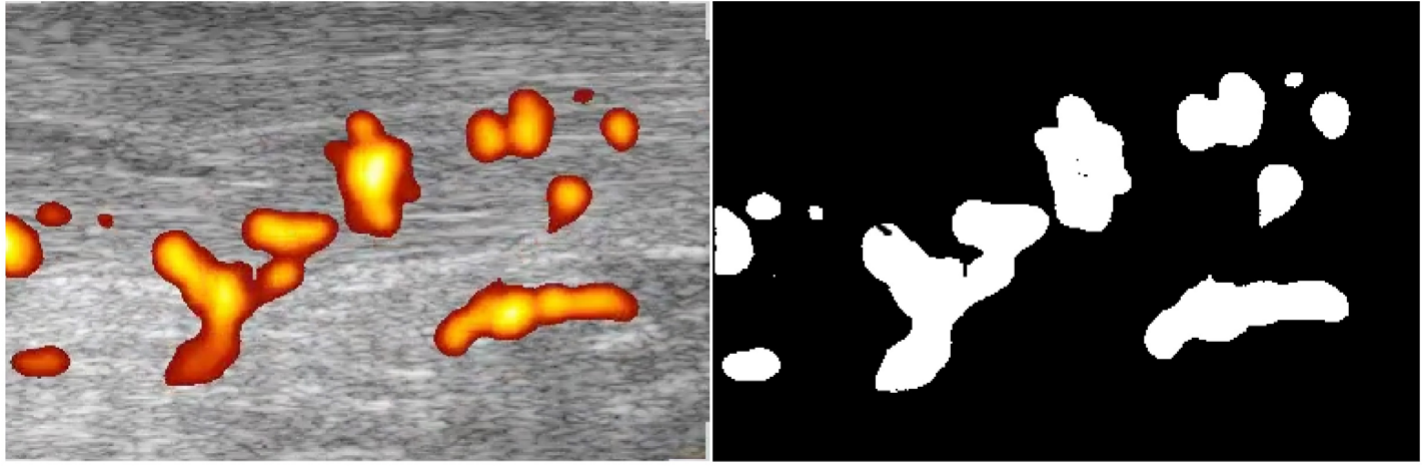
Left, Color box during PDUS of a patient with chronic midportion AT in a sagittal view. Right, Same color box after transformation of color pixels to white pixels to make SAQ possible.

We used Kinovea (Bordeaux, France) software to observe the PDUS video in steps of 0.04 seconds to obtain 3 frames with visually maximum Doppler flow. To measure the surface area of the Doppler flow, we used the program ImageJ version K 1.45 (National Institutes of Health, Bethesda, MD). The area directly around the color box was selected, and the outer part was cleared. Predefined transformations were applied in this study to limit the noise close to 0: threshold color, black and white; hue, 255; saturation, 165; and brightness, 250. After transformation, color pixels of blood vessels were shown as white pixels with a value of 255, and noncolor pixels were shown as black pixels with a value of 0. The number of white pixels was subsequently determined by creating a pixel histogram. The number of white pixels was divided by the total number of pixels in the color box (99,119 pixels) to measure the percentage of displayed blood vessels. This percentage was determined for the 3 frames, and the highest percentage was selected for the analysis. The transformation of the Doppler flow using the SAQ method is illustrated in Figure 1. Both observers analyzed their own PDUS videos and remained blinded to the results from the other observer.

#### *Analysis Using the Modified Öhberg score*

The modified Öhberg score is a 5‐point grading scale to score neovascularization in various types of tendinopathy: 0 indicates the absence of Doppler flow; 1+ indicates 1 or 2 neovessels in the Kager fat pad; 2+ indicates 1 of 2 intratendinous neovessels; 3+ indicates 3 or 4 intratendinous neovessels; and 4+ indicates a network of neovascularization with more than 5 intratendinous neovessels.^10^, ^16^, ^24^

### *Statistical Analyses*

Both researchers imported data from the measurements they performed themselves. The normality of the data was checked visually with Q‐Q plots and statistically with the Shapiro‐Wilk test. The correlation between the degree of Doppler flow measured by the SAQ method and modified Öhberg score was analyzed with the Spearman correlation coefficient. The Spearman correlation coefficient was interpreted as a scale from poor to almost perfect correlation (<0.00, poor; 0.00–0.20, slight; 0.21–0.40, fair; 0.41–0.60, moderate; 0.61–0.80, substantial; and 0.81–1.00, almost perfect).^25^ The interobserver reliability of both methods was analyzed first by determining the Spearman ρ correlation coefficient for non–normally distributed data and the Pearson ρ correlation coefficient for normally distributed data. Correlations were controlled visually by constructing a scatterplot. The ICC for interobserver reliability was determined by the 2‐way random‐effect model with absolute agreement. Interpretation of the ICC was as suggested by Portney and Watkins^26^: values of less than 0.5 indicate poor reliability; scores between 0.5 and 0.75 indicate moderate reliability; and scores of greater than 0.75 indicate good reliability. In addition, the SEM and MDD were determined for both methods by the following equations^27^, ^28^:

$$\mathrm{SEM}=\sqrt{\left(\mathrm{MSw}\right);}$$

$$\mathrm{MDD}=\mathrm{SEM}\times 1.96\times \sqrt{2,}$$

Where MSw represents the within‐subject mean square derived from the analysis of variance. We used SPSS Statistics version 24.0 software (IBM Corporation, Armonk, NY) to analyze data, and statistical significance was set at α = .05 (2 tailed). Additionally, we determined the prevalence‐ and bias‐adjusted κ for ordinal data (PABAK‐OS) for the modified Öhberg score using a Web‐based PABAK‐OS calculator (http://www.singlecaseresearch.org/calculators/pabak-os). We performed this test to adjust for chance agreement of the ordinal modified Öhberg score, since the ICC is designed to be used for continuous outcomes. Using the PABAK‐OS, we were able to determine whether it was justified to determine the ICC for the modified Öhberg score.^29^

## Results

### *Patient Characteristics*

Of the first 32 consecutive patients who were included in the RCT, 4 patients did not participate in this cross‐sectional study. Two patients were lost to follow‐up in the RCT, and 2 patients declined to participate in this study part (lack of time). Consequently, 28 patients participated in this cross‐sectional study, which was performed during follow‐up of the RCT. The mean population age was 49.6 years (range, 35–59 years), and the median symptom duration was 56 weeks (range, 10–1040 weeks). The mean VISA‐A score at baseline was 42.8 points (range, 9–73 points). Detailed baseline demographic characteristics are presented in Table 1.

**Table 1:** Baseline Demographic Characteristics of the Study Population (n = 28)

Characteristic	Value
Mean age ± SD, y	49.6 ± 6.4
Sex, n (%)	
Male	16 (57.1)
Female	12 (42.9)
Mean body mass index ± SD, kg/m^2^	26.3 ± 5.0
Affected side, n (%)	
Left	10 (35.7)
Right	11 (39.3)
Bilateral	7 (25.0)
Median duration of symptoms (IQR), wk	56.0 (10.0–104.0)
Mean VISA‐A score ± SD	42.8 ± 15.0
Primary sport, n (%)	
Running	12 (42.9)
Fitness	3 (10.7)
Cycling	3 (10.7)
Hockey/soccer	2 (7.1)
Handball	1 (3.6)
Volleyball	1 (3.6)
Tai Bo	1 (3.6)
Shooting archery	1 (3.6)
Hiking	2 (7.1)
No sport	2 (7.1)
Activity level, n (%)	
Professional	0 (0)
Competitive	5 (17.9)
Recreational	23 (82.1)

IQR indicates interquartile range.

### *Ultrasound Assessments*

The mean color fraction of the SAQ method ranged from 9.0% to 10.9% for both observers (mean ± SD: observer 1, 9.0% ± 6.6%; observer 2, 10.9% ± 6.8%). The lowest observed color fraction was 0.4%, and the highest was 27.2%. The median of the modified Öhberg score (0–4+) ranged from 3.0 to 3.5 for both observers (observer 1, 3.0 [interquartile range, 1.0]; observer 2, 3.5 [interquartile range, 2.0]). The range was 1+ to 4+ for both observers.

### *Correlation Between the SAQ Method and Modified Öhberg Score*

**Figure 2:**
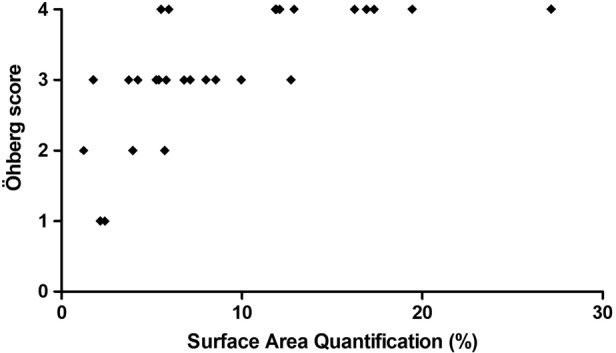
Scatterplot showing the correlation between the modified Öhberg score and SAQ. Note the ceiling effect in the Öhberg 4+ group, in which the SAQ ranges between 5.2% and 27.2%.

The Spearman correlation coefficient between the SAQ method and the modified Öhberg score showed a substantial correlation for both observers. The correlations were found to be 0.76 for observer 1 and 0.62 for observer 2. The SAQ outcomes for the modified Öhberg 4+ group ranged from 5.5% to 27.1% for observer 1 and from 5.2% to 27.2% for observer 2 (Figure 2).

### *Intraclass Correlation Coefficient for Interobserver Reliability*

The ICC of the SAQ method was found to be 0.81 (95% confidence interval [CI], 0.58–0.91), whereas it was 0.64 (95% CI, 0.35–0.81) for the modified Öhberg score. The ICCs, SEMs, and MDDs are reported in Table 2. The PABAK‐OS for the modified Öhberg score was found to be 0.33 (95% CI, 0.17–0.50).

**Table 2:** Intraclass Correlation Coefficients for SAQ Method and Modified Öhberg Score

Method	ICC (95% CI)	*P*	SEM	MDD	n
ICC SAQ	0.81 (0.58–0.91)	<.001	2.88%	7.99%	28
Modified Öhberg score	0.64 (0.35–0.81)	<.001	0.55 points	1.53 points	28

The correlation for an individual measurement method was significant below the .05 level (2 tailed). Note that the SAQ method ranges from 0% to 100%, and the modified Öhberg score ranges from 0 to 4+ points. The SEM and MDD are therefore presented as a percentage for the ICC of the SAQ method and as a number for the modified Öhberg score.

## Discussion

To our knowledge, this was the first study to investigate the reliability of the SAQ method in midportion AT and to compare it with the most widely used quantification method (modified Öhberg score). We showed that the SAQ method is a reliable measurement tool in a research setting to evaluate the degree of US Doppler flow in patients with chronic midportion AT. According to our findings, the SAQ method is not superior to the modified Öhberg score for quantifying neovascularization. There are, however, advantages to the SAQ method, since it is a more quantitative method and overcomes the ceiling effect of the modified Öhberg score by differentiation between high amounts of Doppler flow.

Surface area quantification is a method to quantify the fraction of color pixels during a PDUS examination. We found good interobserver reliability for the SAQ method (ICC, 0.81) compared to moderate interobserver reliability for the modified Öhberg score (ICC, 0.64). Similar findings for the ICC of the modified Öhberg score were published by Risch et al,^19^ in which moderate absolute interobserver agreement (0.64–0.80) was also observed. Sengkerij et al^10^ and Watson et al^12^ observed considerably higher ICCs for the modified Öhberg score (0.85 and 0.86, respectively). In all studies, measurements were determined by experienced radiologists or sports and exercise medicine consultants. One major difference between the studies was that the modified Öhberg scores were determined on the basis of US recordings in the study performed by Risch et al,^19^ whereas the US examinations were performed by the observers themselves in the other 2 studies. This indicates that agreement between observers is not dependent solely on the experience of the observer performing the US examination but also on the observer interpreting the findings. Since the SAQ method selects multiple frames with visually maximum Doppler flow and provides an objective percentage for the color fraction, interpretation of the US recordings that can cause interobserver variability will play a minor role.

We have demonstrated that there is a substantial correlation between the SAQ method and the modified Öhberg score (Figure 2). A perfect correlation was not expected, since the modified Öhberg score is limited to a maximum score of 4, whereas the SAQ method can detect any amount of Doppler flow. The modified Öhberg score is therefore less applicable when there is a high degree of Doppler flow present, as the modified Öhberg 4+ subgroup will represent a wide variation in the degree of Doppler flow. This is called the ceiling effect, in which an extra amount of Doppler flow no longer has an effect on the Öhberg score. This ceiling effect was shown in our study for the modified Öhberg 4+ group (range for the SAQ method, 5.2%–27.2%). This could also be an explanation of why the degree of Doppler flow is to date only weakly related to clinical severity, and future research should therefore focus on the correlation between the SAQ measurement and clinical severity.^13^, ^15^, ^18^

The ICC does not provide a measure of the precision: ie, the difference in individual patients. The precision of a measurement can be determined by calculating the SEM, which reflects the boundaries around the true score of the individual and is largely independent of the variance between patients.^28^ The SEM for the SAQ method was 2.9% in this study. This indicates that when a color fraction of 11.0% is be found, the true value of the observation would be between 8.1% and 13.9%. It is difficult to compare this SEM with the SEM of the modified Öhberg score, since this is an ordinal score ranging from 0 to 4+, whereas the SAQ method ranges from 0% to 100%. The SEM of the modified Öhberg score was 0.55. An observed Öhberg score of 2 would therefore have a true value between 1.45 and 2.55.

The MDD is calculated from the SEM and reflects the threshold for the change within a patient that can be considered a real change.^28^ This is relevant to determine, since these kind of quantification methods will be primarily used during follow‐up to monitor treatment responses. The MDD for the SAQ method was found to be 8.0%. This indicates that in the same patient with a color fraction of 11.0%, a value of less than 3.0% or greater than 19.0% during follow‐up would indicate a real decrease or increase in the color fraction, respectively. The MDD of the modified Öhberg score was found to be 1.5. When an observed Öhberg score of 2 is found in a patient, a value of less than 0.5 or greater than than 3.5 during follow‐up would indicate a real change.

This study had some methodological limitations. First, we chose a standardized size of the color box to only detect intratendinous and peritendinous neovascularization in the Kager fat pad in most of the patients. In a case of a relatively small Achilles tendon, deeper structures such as the posterior tibial artery tibialis could be included in the color box. The presence of this artery was not discussed in the standardized protocol. There was 1 patient in whom this artery was present, and a single observer measured the artery in contrast to the other observer. Determination of the modified Öhberg score was not affected by the presence of the artery in this case. The reliability of the SAQ could potentially be further improved if the posterior tibial artery is recognized and not included in the color box during the US examination. We think that adding this to the SAQ protocol is most appropriate, since the posterior tibial artery is part of the normal vascular structure, and it is not a result of the process of neovascularization. Second, we have not determined intraobserver reliability. In patellar tendinopathy, it was previously demonstrated that day‐to‐day variability in Doppler flow is present; however, in midportion AT, it was shown that the intraobserver reliability of the modified Öhberg score was excellent.^19^, ^30^ Future research should investigate the intraobserver reliability of the SAQ method to verify the diagnostic value of SAQ. Third, ideally a minimum of 3 observers would be involved in a reliability study, since a lack of variability would increase the ICC. We have, however, demonstrated that the SEM indicates fairly good precision. The SEM is, contrary to the ICC, largely independent of variation between patients. We therefore expect that the use of this method would be valid in future research projects. Fourth, US measurements were performed by relatively inexperienced sonographers (1 PhD candidate and 1 research student). Our results demonstrated that the SAQ method is less operator dependent and therefore can be performed well after some practice. Fifth, since the modified Öhberg score is an ordinal scale, the ICC (designed for continuous scales) is not the most appropriate measure for expressing reliability. Therefore, we determined the PABAK‐OS to control for the fact that the chance of agreement is higher in an ordinal scale compared to a continuous scale. We have demonstrated that the PABAK‐OS was indeed lower for the modified Öhberg score, indicating that the ICC does not overestimate the reliability of the modified Öhberg score. Sixth, we chose to refrain from a power analysis, since data for this novel measurement tool were not available when designing this study, and post hoc power analyses are discouraged.^31^ Last, the SAQ method is currently not available as a measurement function on US machines, and computers and (free) software packages have to be used to determine the value of the SAQ. Since we used a relatively easy method to calculate the value of the SAQ, we expect that this function could be implemented easily on US machines via a real‐time application once it has shown its clinical relevance in future studies through estimating the prognosis or personalized treatment based on SAQ scores.

In conclusion, to our knowledge, this was the first study to evaluate the diagnostic value and reliability of measuring the surface area of Doppler flow in patients with chronic midportion AT. This study demonstrates that SAQ has similarly good reliability as the modified Öhberg score. Surface area quantification, however, overcomes disadvantages of the modified Öhberg score, of which the ceiling effect in the modified Öhberg 4+ category is most important. These findings could inspire medical experts to use the SAQ method for research purposes to determine the degree of US Doppler flow quantitatively in patients with chronic midportion AT. Ultimately, treatment responses of interventions acting on neovascularization could be monitored quantitatively and with good reliability. More research is needed regarding the intraobserver reliability to evaluate the clinical applicability.
